# Intensity Modulated Radiotherapy with Carbon Ion Radiotherapy Boost for Acinic Cell Carcinoma of the Salivary Glands

**DOI:** 10.3390/cancers13010124

**Published:** 2021-01-02

**Authors:** Maximilian P. Schmid, Thomas Held, Kristin Lang, Klaus Herfarth, Juliane Hörner-Rieber, Semi B. Harrabi, Julius Moratin, Christian Freudlsperger, Karim Zaoui, Jürgen Debus, Sebastian Adeberg

**Affiliations:** 1 Heidelberg Ion-Beam Therapy Center, Department of Radiation Oncology, Heidelberg University Hospital, 69120 Heidelberg, Germany; maximilian.a.schmid@meduniwien.ac.at (M.P.S.); thomas.held@med.uni-heidelberg.de (T.H.); kristin.lang@med.uni-heidelberg.de (K.L.); klaus.herfarth@med.uni-heidelberg.de (K.H.); juliane.hoerner-rieber@med.uni-heidelberg.de (J.H.-R.); semi.harrabi@med.uni-heidelberg.de (S.B.H.); juergen.debus@med.uni-heidelberg.de (J.D.); 2 Department of Radiation Oncology—Comprehensive Cancer Center, Medical University of Vienna, 1090 Vienna, Austria; 3 Heidelberg Institute of Radiation Oncology (HIRO), 69120 Heidelberg, Germany; 4 Clinical Cooperation Unit Radiation Oncology, German Cancer Research Center (DKFZ), 69120 Heidelberg, Germany; 5 Department of Oral and Maxillofacial Surgery, University Hospital Heidelberg, 69120 Heidelberg, Germany; julius.moratin@med.uni-heidelberg.de (J.M.); christian.freudlsperger@med.uni-heidelberg.de (C.F.); 6 Department of Otorhinolaryngology, University of Heidelberg, 69120 Heidelberg, Germany; karim.zaoui@med.uni-heidelberg.de

**Keywords:** particle therapy, C12, head and neck cancer

## Abstract

**Simple Summary:**

Acinic cell carcinoma is a rare disease treated usually by surgery. The role of radiotherapy is controversially discussed. In this retrospective analysis based on 15 patients undergoing postoperative or definitive radiotherapy (intensity-modulated radiotherapy, IMRT) with carbon ion radiotherapy boost leads to excellent results after R1-resection, and is a promising treatment modality in inoperable patients. G1-2 xerostomia, dysgeusia, and trismus were the main reported morbidity symptoms after radiotherapy. Confirmation of the results with larger patient cohorts is needed.

**Abstract:**

*Aim*: to report clinical outcome in patients with acinic cell carcinoma of the salivary glands treated with intensity-modulated radiotherapy (IMRT) and carbon ion radiotherapy (CIRT) boost. *Materials and Methods*: all patients with acinic cell carcinoma of the salivary glands treated at the Heidelberg Ion-Beam Therapy Center were considered for this retrospective analysis. All patients received a CIRT boost with 18–24 Gy radiobiologic effectiveness (RBE)-weighted dose in 3 Gy RBE-weighted dose per fraction followed by IMRT, with 50–54 Gy in 2 Gy per fraction. Disease outcome was evaluated for local (LR), nodal (NR), distant recurrence (DR), and disease-free (DFS) and overall survival (OS). Morbidity was scored based on Common Terminology Criteria for Adverse Events (CTCAE) version 5. Descriptive statistics and the Kaplan-Meier method were used for analysis. *Results*: fifteen patients were available for analysis. Median follow-up after radiotherapy was 43 months. Six patients were treated for primary disease and nine for recurrent disease. Eight patients were treated with radiotherapy for macroscopic disease. Disease recurrence was observed in four patients: 1 LR, 2 NR, and 2 DR; 5-year local control, DFS, and OS were 80%, 52%, and 80%, respectively. No radiotherapy-related G3-5 morbidity was observed. *Conclusion*: In acinic cell carcinoma, IMRT with carbon ion radiotherapy boost leads to excellent results after R1-resection and is a promising treatment modality for definitive treatment in inoperable patients.

## 1. Introduction

Acinic cell carcinoma is a rare malignancy, which typically arises in the parotid gland and comprises approximately 7% of all parotid malignancies [1]. In the majority of cases, surgery is the treatment of choice and the role of radiotherapy as postoperative or definitive treatment is controversially discussed [2]. In accordance to other salivary gland tumors, acinic cell carcinomas are considered rather radioresistant for photon radiotherapy, as shown by the superior disease outcome results of neutron radiotherapy in comparison to photon radiotherapy [3]. Carbon ion radiotherapy is characterized by a similar radiobiologic effectiveness (RBE) as neutron radiotherapy, but with an improved dose profile and treatment planning. First clinical results on carbon ion radiotherapy in overall salivary gland tumors demonstrated promising local tumor control rates and morbidity results [4,5,6]. However, data exclusively for acinic cell carcinoma is missing so far. Here, we report our experience with intensity modulated radiotherapy (IMRT) and carbon ion radiotherapy boost in patients with acinic cell carcinoma.

## 2. Results

### 2.1. Patient Cohort

In total, 15 patients were available for analysis. Median age was 53 years, 10 (67%) female, and 5 (33%) male patients. Six (40%) patients were treated for primary disease and nine (60%) for recurrent disease. A median number of three surgical resections was performed prior to the start of radiotherapy. Eight patients were treated with radiotherapy for macroscopic disease. Nine (60%) patients had T3–4 tumors and five (33%) had nodal involvement. One patient had confirmed distant metastasis at diagnosis. Details are presented in Table 1.

### 2.2. Treatment Characteristics

Radiotherapy treatment characteristics are shown in Table 2. All patients received a carbon ion radiotherapy boost. Median clinical target volume (CTV) 1 measure and median prescribed dose for carbon ion radiotherapy were 106 cc (range: 32–594) and 24 Gy RBE-weighted dose, respectively. Median CTV2 measure was 270 cc (range: 99–943) and comprised in the majority of patients the ipsilateral lymph node levels Ib—III. Median prescribed dose for IMRT was 50 Gy (range: 50–54). The median total prescribed cumulative radiotherapy dose (carbon ion radiotherapy + IMRT) was 74 Gy RBE-weighted dose corresponding to a dose of 80 Gy EQD2 (radiobiological equivalent dose in 2 Gy, α/β = 2).

### 2.3. Clinical Outcome

Median follow-up from initial diagnosis was 99 months (range: 7–470 months) and median follow-up from diagnosis for radiotherapy referral was 43 months (range: 7–130 months). Two patients were lost-to-follow-up 53 and 65 months after radiotherapy. Overall, disease recurrence was observed in four patients: one local recurrence, two nodal recurrences, and two distant recurrences were reported (one simultaneous nodal and distant recurrence). Three patients died (two with nodal disease recurrence, one with systemic progression in primary metastasized disease). The 10-year overall survival (OS) (from initial diagnosis) was 80% (95% CI: 50–93%) and 5-year OS, disease-free (DFS), and local control (LC) were (from diagnosis for radiotherapy referral) was 80% (95% CI: 50–93%), 57% (23–81%) and 80% (20–97%).

The morbidity spectrum before radiotherapy and during follow-up is presented in Table 3. No radiotherapy-related acute and late grade 3–5 morbidity was observed. The predominant morbidity before start of radiotherapy was facial palsy (*n* = 9, 60%) and hearing impairment (*n* = 3, 20%). The predominant morbidity at 6–12 weeks after radiotherapy was xerostomia (*n* = 13, 87%), dysgeusia (*n* = 11, 73%), and trismus (*n* = 10, 67%), whereas facial palsy improved. Comprehensive morbidity reporting 12 months after radiotherapy was available only in a reduced number of patients (*n* = 7) and showed a similar pattern as for 6–12 weeks follow-up but with improvement of dysgeusia. One patient developed an asymptomatic blood-brain-barrier disorder (grade 1).

All patients with disease recurrences were treated by radiotherapy for macroscopic disease. All of these patients had T3–4 disease and two were node positive. No recurrence was observed in patients treated postoperatively for positive resection margins.

**Figure 1:**
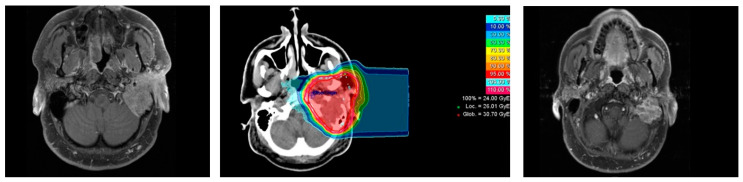
Example of patient with primary acinic cell carcinoma cT4 pN2a cM0; left panel: contrast-enhanced T1-weighted MRI at diagnosis; middle panel: carbon-ion radiotherapy boost plan; right panel: contrast-enhanced T1-weighted MRI at follow-up with in-field local recurrence.

The local recurrence was documented in a patient with cT4 pN2a 53 months after diagnosis (Figure 1). Radiotherapy was performed for primary disease in definitive setting. The local recurrence was classified as in-field recurrence (inside CTV1). The patient received then re-irradiation with carbon-ion radiotherapy only (dose: 51Gy RBE-weighted dose in 3 Gy RBE-weighted dose fractions) and later surgery due to further disease progression and radionecrosis occipitocerebellar. Lung metastases were detected 109 months after initial diagnosis. The patient is currently being observed with regular imaging and is alive with evidence of disease 130 months after radiotherapy.

Nodal recurrences were documented in two previously node negative patients with locally advanced disease (pT3 with R2 resection and rcT3) and were located in the ipsilateral neck inside the CTV2. One patient was treated then by surgery with R0 resection of the nodal recurrence (interval from diagnosis to recurrence: 53 months) and was afterwards lost-to-follow-up and one patient had beside the nodal recurrence simultaneous lung metastases (interval from diagnosis to recurrence: 17 months) and died 3 months after the detection of the recurrence.

One distant recurrence, in addition to the above-mentioned patient, was observed in a patient with stage cT3 cN2b disease, who developed a multifocal meningeal recurrence 8 months after diagnosis and died 12 months later. The patient with primary metastasized disease (pT4, pN1, cM1 (bone and lungs), Pn1, L1, V1, R1) died 7 months after diagnosis.

## 3. Discussion

Literature on acinic cell carcinoma is scarce and is based on either small single center cohort analyses or larger heterogeneous national databases. Acinic cell carcinoma is characterized by slow tumor growth, high long-term survival, and a tendency towards late recurrences. Management of acinic cell carcinoma is predominately surgical with 10-year OS of approximately 90%. The majority of patients in these cohorts have stage T1–2 tumors, no regional lymph node metastasis and approximately 40–50% received adjuvant radiotherapy [7,8]. Locally advanced stage, tumor size, high grade, close, or positive margins, lymph node metastasis, perineural or lymphovascular space invasion and higher age are considered as risk factors to select patients for adjuvant treatment, but the benefit of adjuvant radiotherapy is unclear. North et al. [9] concluded based on 744 patients with intermediate grade salivary gland carcinomas (19% with acinic cell carcinoma) a significant improvement by adjuvant radiotherapy in patients with positive resection margins, whereas Andreoli et al. [10] reported in 1241 patients with acinic cell carcinoma no difference in overall survival if adjuvant radiotherapy is performed or not. Definitive radiotherapy is generally restricted to patients unfit, refusing surgery [11] or if surgery with clear margins necessitates mutilating procedures. Literature on definitive radiotherapy in acinic cell carcinoma is even less available than for adjuvant radiotherapy and is mainly presented as part of outcome results of overall salivary gland carcinomas [4,5,6]. Mendenhall reported 10 yr OS of 34% (5 yr OS: ~50%) in 64 patients with inoperable salivary gland carcinomas (majority adenoid-cystic carcinomas; 2% with acinic cell carcinoma) after a median dose of 74 Gy with (photon-based) radiotherapy only [12].

To the best of our knowledge, this is the first report on carbon ion radiotherapy exclusively in acinic cell carcinoma. Our small cohort (*n* = 15) comprised a high-risk group with locally advanced stage (T3–4: *n* = 9, 60%), positive lymph nodes (*n* = 5, 33%), locally recurrent disease (*n* = 9, 60%), no surgery (for primary or recurrent disease, *n* = 6, 40%) or R2-resection (*n* = 2, 13%) and presence of organ metastasis (*n* = 1, 7%). Neskey et al. [13] reported in patients with locally recurrent disease a 9-fold higher risk for the development of another local recurrence in comparison to no previous history of acinic cell carcinoma. In our cohort, a median number of three previous surgical resections was performed prior to the referral to radiotherapy. Five-year OS in our cohort was 80% and 5-year DFS after radiotherapy was 52%, which seems to underline the selection of high-risk patients. An overview of clinical outcome and presence of risk factors in modern series is summarized in Table 4.

**Figure 2:**
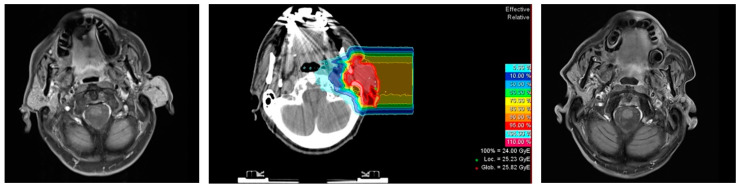
Example of patient with recurrent acinic cell carcinoma rcT2 cN0 cM0; left panel: contrast-enhanced T1-weighted MRI at diagnosis; middle panel: carbon-ion radiotherapy boost plan; right panel: contrast-enhanced T1-weighted MRI at follow-up with local control.

Notably, despite absence of surgery in 40% of our patients, 5 yr DFS and LC is comparable to the high-risk sub-groups in the surgical (+/− adjuvant radiotherapy) series. Only one local recurrence was observed in the overall cohort and no local, nodal, or distant recurrence was observed after R1-resection. It seems as if the performance of high dose radiotherapy including carbon ion radiotherapy boost can effectively eradicate microscopic disease after R1-resection and even macroscopic disease without surgery (or after R2-resection). An example is provided in Figure 2.

Two nodal recurrences were documented, in previously node negative patients. In accordance, Grasl et al. reported 15% (*n* = 4) occult lymph node metastasis after elective neck dissection in 27 patients with acinic cell carcinoma of the parotid gland [14]. The administration of the 50 Gy elective dose with photon based IMRT was obviously not sufficient to prevent nodal recurrence in these two patients.

Previous studies on carbon ion radiotherapy in salivary gland carcinomas point towards similar outcome with high local control and survival. In the COSMIC trial (*n* = 53, 89% adenoid-cystic carcinomas, 57% stage T4) 3-yr LC was 82% and 3-yr OS was 78% [5]. In 40 patients with salivary gland carcinomas other than adenoid cystic carcinomas (majority mucoepidermoid carcinoma, 3 patients with acinic cell carcinoma) 3-yr LC was 82% and 3-yr OS was 73% [6]. Hayashi reported in 69 patients with salivary gland carcinomas (majority adenoid cystic carcinoma, 5 patients with acinic cell carcinoma) treated with carbon-ion radiotherapy alone 5-yr LC and OS of 74% and 82%, respectively [4].

Morbidity outcome is comparable to other carbon ion radiotherapy studies in salivary gland carcinomas. Substantial baseline morbidity as a result of the destruction by the tumor and multiple previous surgeries was present before start of radiotherapy. Xerostomia, dysgeusia, and trismus were the main reported symptoms after radiotherapy. No radiotherapy-related grade 3–5 morbidity was observed.

The main limitations are the retrospective study design and the small patient cohort. Long term morbidity results have to be interpreted with caution due to the limited number of patients with comprehensive data on morbidity 12 months after radiotherapy. Nevertheless, the new perspective on acinic cell carcinoma treated with carbon ion radiotherapy is unique and appears valuable despite the small sample size. Confirmation of the results with larger patient cohorts is needed.

## 4. Materials and Methods

### 4.1. Patients

All patients with acinic cell carcinoma of the salivary glands treated between 2010 and 2018 at the Heidelberg Ion-Beam Therapy Center (HIT) were considered for this retrospective analysis. Inclusion criteria were any age, biopsy-proven primary or locally recurrent disease and performance of carbon ion radiotherapy. All patients were staged with MRI for assessment of local disease and CT for assessment of metastatic disease. Radiotherapy was applied as postoperative treatment after surgery in patients with positive resection margins or residual macroscopic tumor or as definitive treatment in patients refusing surgery, with inoperable tumors or with locally recurrent tumors after multiple resections.

### 4.2. Radiotherapy

All patients received a carbon ion radiotherapy boost followed by IMRT. The reversed boost sequence is an in-house protocol for head-and-neck tumors undergoing particle therapy and is applied to reduce particle range uncertainties, e.g., due to alternating mucosa swelling in the second half of the treatment. Patients were immobilized with individualized thermoplastic head masks. Target definition was based on native/contrast enhanced CT scans fused with contrast-enhanced MRI. The CTV for carbon ion radiotherapy (CTV1) consisted of the gross tumor volume (GTV)/salivary gland/tumor bed plus an individual margin based on the oncologic setting and the CTV for IMRT (CTV2) encompassed the CTV1 plus regional lymph node levels. Additional 3 mm margins were used to generate the planning target volumes (PTV) in IMRT planning and 2 mm for carbon ion plans. The difference in CTV-PTV margin for carbon ion radiotherapy and IMRT is based on the lower uncertainties and movements at the level of the base of the skull for carbon ion radiotherapy in contrast to the neck with IMRT. Based on the nodal status and the presence of a macroscopic tumor 18–24 Gy RBE-weighted dose were prescribed in daily fractions of 3 Gy RBE-weighted dose to CTV1 and 50–54 Gy with 2 Gy per fraction were prescribed to CTV2 corresponding to a cumulative dose of 76.5–80 Gy EQD2 (radiobiological equivalent dose in 2 Gy, α/β = 2) [15]. Syngo PT Planning (Siemens, Erlangen, Germany) was used for carbon ion radiotherapy treatment planning and TomoTherapy^®^-Planning Station (Accuray, Sunnyvale, CA, USA) for IMRT treatment planning. Carbon ion radiotherapy was performed at the HIT with active raster scanning using 1–2 beams. Details of carbon ion radiotherapy are described elsewhere [16]. IMRT was performed as helical IMRT with daily image guidance (TomoTherapy^®^, Accuray, Sunnyvale, CA, USA).

Follow-up including clinical examination, MRI of head and neck was performed for 2 years in 3 months intervals starting 6–12 weeks after end of treatment, then in 6 months intervals until the fifth year after treatment and then annually thereafter. Additionally CT of chest and abdomen was performed in 12-month intervals.

### 4.3. Data Collection and Study Endpoints

The study was approved by the Ethics committee of the University of Heidelberg (S-421/2015). Due to the retrospective study design, no study specific informed consent was necessary according to the local ethical guidelines. Data on patient, tumor and treatment characteristics was retrospectively collected from the medical records. Data cutoff refers to 21 July, 2020. Morbidity results were derived from the standardized follow-up forms (including Common Terminology Criteria for Adverse Events (CTCAE) classification) used in clinical routine and were supplemented by entries or comments in the medical records; scoring was then adapted for CTCAE version 5. Disease outcome was evaluated for local recurrence, nodal recurrence and distant recurrence. OS was defined by death from any cause. DFS was defined as any disease recurrence or death from any cause. Time-to-event intervals were calculated from the date of diagnosis until the respective event. Patients without events were censored at the date of last follow up; 95% confidence intervals (CI) were calculated for time-to-event estimates. Descriptive statistics (Excel, Microsoft) and the Kaplan–Meier method (SPSS version 26) were used for analysis.

## 5. Conclusions

In acinic cell carcinoma, IMRT with carbon ion radiotherapy boost leads to excellent results as postoperative treatment after R1 resection and is a promising treatment modality for definitive treatment in inoperable patients.

## Figures and Tables

**Table 1:** General patient, tumor and treatment characteristics.

Parameter	*n*
Total number of patients (*n*)	15
Median age at diagnosis in years (range)	53 (19–79)
Gender (*n*)	
male	5
female	10
Local tumor status at diagnosis (*n*)	
primary	6
recurrent	9
Median number of previous surgeries (range)	3 (0–6)
Location (*n*)	
parotid	14
submandibular	1
T-stage (*n*)	
T1	3
T2	3
T3	5
T4	4
N-stage (*n*)	
N0	10
N+	5
M-stage (*n*)	
M0	14
M1	1
Radiotherapy setting (*n*)	
definitive	6
postoperative	9
Resection status	
R0	0
R1	7
R2	2

**Table 2:** Radiotherapy characteristics (*n* = 15).

**Carbon Ion Radiotherapy**	**metric**
Median GTV in cm^3^ (range) *	37 (15–182)
Median CTV1 in cm^3^ (range)	106 (32–594)
Median prescribed single dose in Gy RBE-weighted dose (range)	3 (3–3)
Median prescribed total dose in Gy RBE-weighted dose (range)	24 (18–24)
Number of beams (*n*)	
1	7
2	8
**IMRT**	
Median CTV2 in cm^3^ (range)	270 (99–943)
Included lymph node levels	
Ib-III ipsilateral (*n*)	10
Ib-V ipsilateral (*n*)	2
Ib-IV ipsilateral + Ib-III contralalteral (*n*)	1
Ib-III bilateral (*n*)	1
Ib-IV bilateral (*n*)	1
Median prescribed single dose in Gy (range)	2 (2–2)
Median prescribed total dose in Gy (range)	50 (50–54)
Median cumulative dose in Gy RBE-weighted dose	74 (72–74)

* *n* = 5.

**Table 3:** Morbidity.

Morbidity	G1 (*n*)	G2 (*n*)	G3 (*n*)	Overall (*n*, %)
Start of radiotherapy (*n* = 15)				
Xerostomia	2	0	0	2 (13%)
Dysphagia	1	0	0	1 (7%)
Trismus	0	2	0	2 (13%)
Facial palsy	4	5	0	9 (60%)
Hearing impairment	1	0	2	3 (20%)
Pain	2	0	0	2 (13%)
6–12 weeks follow-up (*n* = 14)				
Mucositis	0	0	0	0 (0%)
Skin	5	0	0	5 (36%)
Xerostomia	12	1	0	13 (93%)
Dysphagia	2	1	0	3 (21%)
Trismus	2	8	0	10 (71%)
Facial palsy	3	2	0	5 (36%)
Hearing impairment	2	0	2	4 (29%)
Pain	5	0	0	5 (36%)
Dysgeusia	9	2	0	11 (79%)
Fatigue	2	0	0	2 (14%)
Alopecia	4	1	0	5 (36%)
Lymph edema	3	1	0	4 (29%)
Maximum toxicity after 12 months follow-up (*n* = 7)				
Mucositis	0	0	0	0 (0%)
Skin	0	0	0	0 (0%)
Xerostomia	4	2	0	6 (86%)
Dysphagia	1	0	0	1 (14%)
Trismus	2	4	0	6 (86%)
Facial palsy	2	2	0	4 (57%)
Hearing impairment	1	0	2	3 (43%)
Pain	1	0	0	1 (14%)
Dysgeusia	1	1	0	2 (29%)
Fatigue	2	0	0	2 (29%)
Alopecia	0	0	0	0 (0%)
Lymph edema	2	1	0	3 (43%)
Brain	1	0	0	1 (14%)

**Table 4:** Literature overview.

Study	Number of Patients	Risk Factors	Treatment	Outcome
Zenga et al. 2018 [7]	45	T3–4: 18%; N+: 7%; R1: 16%	100% surgery + 49% adjuvant RT	overall: 5 yr OS: 93; 5 yr DFS: 89%; high risk patients: 5 yr DFS: ~58%
Scherl et al. 2018 [8]	2362	T3–4: 9%; N+: 8%; R1: n.a.	100% surgery + 42% adjuvant RT	overall: 5 yr OS: 88%; pT3–4: 5 yr OS: 69%, pN+: 5 yr OS: 56%
Neskey et al. 2013 [13]	155	T3–4: 12%; N1:n.a., N2–3:4%; R1: 34%	100% surgery + 50% adjuvant RT	overall: 5 yr OS: n.a., LC: 80%; pT3–4: 5 yr OS: ~65%
Andreoli et al. 2012 [10]	969	T3–4: 13%; N+: n.a.; R1: n.a.	100% surgery + 34% adjuvant RT	no difference between surgery alone versus surgery + radiotherapy
Present study 2020	15	T3–4: 60%, N+: 33%; R+: 60%	60% surgery + 100% RT	overall: 5 yr OS: 80%, 5yr LC: 80%, 5 yr DFS: 57%

n.a.: not available, OS: overall survival, DFS: disease-free survival, LC: local control.

## Data Availability

The datasets used and/or analyzed during the current study are available from the corresponding author on reasonable request.
